# Supplementary material for: Are carbon emissions trading and green financial instruments synergistic? -Comprehensive quantitative research based on content analysis
Source: PLoS One. 2024 Mar 7;19(3):e0298601. doi: 10.1371/journal.pone.0298601 (PMC10919601; doi:10.1371/journal.pone.0298601)
Supplement: S1 Data — (ZIP) [file pone.0298601.s001.zip › Data/地级市Z_F工作报告.zip!/║ú─╧/║ú┐┌╩╨/└┤╫╘╣1⁄2╓┌║┼í╛┬φ┐╦╩2╛▌═°í┐_2017.docx]

各位代表：

现在，我代表海口市人民政府向大会报告工作，请予审议，并请各位政协委员和列席人员提出意见。

一、2016年工作回顾

一年来，我们紧密团结在以习近平同志为核心的党中央周围，全面贯彻党的十八大和十八届历次全会精神，在省委省政府和市委的坚强领导下，在市人大、市政协的监督支持下，坚决扛起省会城市的“三个担当” “五个带头”作用，坚持新发展理念，以供给侧结构性改革为主线，以“双创”为总抓手和动力，有效应对经济持续下行压力和强台风等自然灾害侵袭，经济社会各项事业取得显著成效，圆满完成了年初确定的主要目标任务，实现了“十三五”良好开局。

2016年，全市地区生产总值完成1257.7亿元，增长7.7%，增速高于全国、全省。固定资产投资完成1271.7亿元，增长25.7%，总量和增速均为全省第一，增速继续位居全国35个大中城市前列。全口径公共预算收入完成330.9亿元，增长23.7%，其中地方一般公共预算收入完成115.5亿元，同口径增长11.2%。社会消费品零售总额653. 9亿元，增长9.8%。城乡常住居民人均可支配收入达26447元，增长8.2%。居民消费价格指数上涨3%，城镇登记失业率0.9%。省下达的节能减排目标任务全面完成。地区生产总值、固定资产投资、全口径公共预算收入在全省占比分别达31.1%、33.9%和30.6%，省会城市首位度进一步提高。

一年来，主要抓了以下七个方面的工作：

（一）全面推进“双创”攻坚，城市品质有效提升

市政基础设施日益完善。共启动“双创”项目256个，完成投资20.2亿元，筑牢了民生“里子”，撑起了城市“面子”。改造小街小巷1576条、老旧市政主干道路13条，升级农贸市场39个，打造“15分钟便民生活圈”39处、特色餐饮街（区）10条。改造城市积水点7处，建成排涝泵站6个。集中对国兴大道、椰海大道等主要道路和西海岸带状公园、万绿园等23个项目进行景观改造提升，完成125条道路、22个街边街心绿地绿化补植。民航航线俯视区生态修复完成设计编制，18个全域旅游镇墟改造全部进场施工，高铁高速沿线今年4月有望完成整体景观提升。海口宜居竞争力在中国社会科学院2016年评比的289个城市中排名第五。城市治理持续加强。成立城市警察支队，推行“公安+城管”联合执法，实现了行政处罚到刑事处罚无缝对接。建立辖区政府主导，商务、工商、质监、食药监等部门联合执法的监管模式，农贸市场管理水平全国领先。建成区主要道路机扫率达100%，海口成为全国第一个主城区全面实现环卫一体化改革的城市。“城市管家”“公益海口”“诚信海口”三大网络服务平台投入运营，“12345”热线服务质量有效提升，全年受理各类诉求33.4万件，办结率达97.2%。城市公共治理指数首次进入全国省会城市前十。社会总动员能力和市民素质大幅提升。凡人善举不断涌现，崇德向善、邻里互助蔚然成风，涌现了“全国文明家庭”李朵家庭、“中国好人”盘丽芬等一批先进典型，全市注册志愿者25万人，被《人民日报》点赞为“志愿者之城”。全市各级干部夜以继日，忘我工作，以辛苦指数换取群众满意指数、城市发展指数和平安幸福指数。特别是我们动员全社会众志成城、团结一心，有效应对“莎莉嘉”等强台风，灾后一天内恢复全市生产生活秩序。“创文”在全国参评省会城市中排名前列；“创卫”顺利通过国家暗访测评、位居参评省会城市第一，近期又得到国家技术评估专家组的高度肯定，市民和游客满意度高达98.7%。

（二）着力强化需求拉动，经济运行稳中向好

项目投资高位增长。服务社会投资百日大行动项目160个全部开工建设，86个省重点项目完成投资665亿元、完成计划任务的143%，有望获得省突出贡献奖。积极引入社会资本参与市政设施、公共服务等领域建设，南渡江引水工程等6个PPP项目入选财政部示范项目。海秀快速路、定海大桥建成通车，桂林洋热带农业国家公园、美兰机场二期、马村港三期、地下综合管廊等项目加快建设，美兰临空产业园、新海生态临港新城等一批项目启动前期工作。招商引进项目198个、协议投资1594亿元，落地率达51%。特别是在省委、省政府的大力支持下，成功引进万达文化旅游城，单体投资超过500亿元，建成后将成为海南国际旅游岛的标志性项目。消费市场繁荣活跃。接待国内外过夜游客1329.2万人次，实现旅游总收入191.8亿元，分别增长8.5%、13.6%；房地产销售面积432.6万平方米、销售额391亿元，分别增长15.9%、31.9%；“冬交会”签约订单36.9亿，增长4.8%。启动首届“互联网+”新型消费活动，推动线上+线下“一站式”服务，林安物流信息交易中心落成开业，日月广场、新城吾悦、万达广场等一批大型商业综合体开业运营。对外贸易稳步拓展。开展服务贸易创新发展试点，承接省里8大类47项创新举措，海马汽车出口伊朗，齐鲁制药注射剂成功打入欧美高端市场，飞机融资租赁总货值完成26.3亿元、汽车整车进口450辆，进出口总额完成39.2亿美元。

（三）深入推进供给侧结构性改革，新旧动能加快转换

围绕中央“三去一降一补” 要求和省委省政府“十二大产业” 部署，出台医疗健康、影视会展等15项产业政策，精准培育发展新动能。以旅游业为龙头的现代服务业快速发展。旅游业转型升级。“点线面”推进全域旅游，东寨港红树林、冯小刚电影公社、骑楼老街等景点景区渐成热点，长影环球100、观澜湖水世界等项目加快建设，首届海南国际旅游岛三角梅花展成功举办，乡村民宿、影视娱乐、时尚文化等旅游新业态不断涌现，获评中国“互联网+旅游”十大优秀城市。互联网产业蓬勃发展。阿里巴巴“南海云”建成运行，复兴城创新创业园互联网企业聚集度排名全国前列，石山互联网农业小镇创造全国经验，互联网企业新增近千家，营业收入155亿元、增长23%，占全省48.4%。被列为“宽带中国”示范城市。金融业进一步发展壮大。民生银行进驻，落户银行增至23家，金融机构存、贷款余额分别比年初增长919.6亿元、671.6亿元，保险机构保费收入增长10.7%。实现金融业增加值152.7亿元、增长16.7%，占全省54.5%。医疗健康产业发展势头良好。复旦大学附属华山医院海口友好合作医院开诊，上海第六人民医院海口骨科与糖尿病医院落地筹建，省肿瘤医院、恭和苑、颐养公社等医养结合稳步发展，获批中医养生服务业准入试点城市。房地产去库存成效明显。去库存周期降至15个月，低于全国和全省平均水平，房价基本保持稳定。文化体育产业发展提速。中国（南方）足球训练基地加快建设，爱奇艺创意中心、阿里巴巴文化娱乐集团南方总部等100多家企业成功入驻海口文化产业园。会展业快速发展。成功举办中国生态文明论坛海口年会、第23届中国国际广告节等规模以上会展活动238场，会展综合收入达80亿元，占全省35%，连续五年获得“中国会展名城”大奖。新型工业量质同升。全年引进工业项目17个、签约额92亿元，康迪电动汽车、创志智能机器人等项目当年引进、当年开工。先声药业、灵康药业等项目加快建设，医药行业产值达154.7亿元、增长11.8%。齐鲁制药、金盘电气荣获首届海南省政府质量奖，高新技术企业总数达149家，占全省75%。现代农业发展步伐加快。推出火山石斛、云龙淮山等十大农业品牌，永兴黄皮获国家地理标志证明商标。国家、省级农业龙头企业达45家，省级现代农业示范基地（园区）9家。

（四）持续深化改革开放，发展活力明显增强

“多规合一”改革加快推进。将主体功能区、城镇体系、土地、海洋、林地、生态等六类空间规划合成一个规划，形成《海口市总体规划（空间类2015-2030）》成果，建立了发展目标、指标和坐标对应的“一张蓝图”，化解矛盾空间852平方公里，为推进行政审批改革、管理体制改革和资源要素市场化配置改革创造了有利条件。“放管服”改革深入开展。市级行政审批服务事项减少70%，审批环节消减37%，审批时间压缩50%；实行多部门并联审批、联合审批和联合验收，“极简审批”模式得到国务院第三次大督查的充分肯定，点赞海口“找到了企业发展与政府监管的最大公约数”。实施“五证合一、一照一码” 登记制度改革，“先照后证” 全面落实。全年新增市场主体5.3万户、新增注册资本（金）4774亿元，分别增长33.3%、402.8%，市场主体占全省73%。财税体制、国资国企、医疗卫生等改革取得重要进展。“营改增”全面实施；完成海口港与洋浦港小铲滩码头的资源整合，新海一期资产证券化获得国家证监会批准；全面启动国家第四批公立医院改革试点，稳步推进分级诊疗，市域内11家公立医院全面取消药品加成；积极承接农垦改革，设立全国首个以“居”命名的社会自治组织——东昌居；成立市不动产登记中心，不动产登记4.7万宗。

不断扩大对外开放。围绕打造国家“一带一路”战略支点城市，深化区域合作，《海澄文一体化区域基础设施规划》编制完成，《海口国家新区总体方案》已上报国务院；积极推动琼州海峡经济带建设，深化与湛江在发展规划、交通港航、安全生产、反恐维稳、旅游、菜篮子等领域的务实合作。着力构建互联互通体系，海口港内外贸海运航线达30条，集装箱吞吐量140万标箱、增长10.7%；美兰机场国际航线达25条，客流量1880万人次、增长16.3%。第四届APEC蓝色经济论坛、首届“2016全球旅游目的地盛典”、第四届观澜湖世界明星赛等重大活动成功举办，新增国际友城3个，海口对外开放度和影响力进一步提升。

（五）严守生态保护红线，扎实开展“六大专项整治”

重拳打违控违。全年拆除违法建筑337万平方米，防违控违53万平方米。大力整治城乡环境脏乱差。农村生活垃圾无害化处理率达到95%，全市576个“三无”小区完成集中治理；新建垃圾转运站35座、厕所131座，餐厨垃圾及粪渣无害化处理厂建成投入使用。启动城镇内河（湖）水污染治理。率先推行“河长制”，集中开展31个水体治理，主城区水环境功能基本达到国家卫生城市标准。强化大气污染防治。建立环境空气质量预警预报系统，开展北部湾大气联防联治，淘汰黄标车、老旧车1.4万辆，空气质量连续七年在全国74个重点城市中名列第一。加强耕地修复与治理，土壤环境质量保持总体稳定。持续推进生态修复和湿地保护。“绿化宝岛”造林绿化2万亩，查处南渡江非法采沙348宗。完成三江湿地公园规划设计，红树林生态环境持续向好，国家一级保护动物——黑脸琵鹭消失7年后重回东寨港。海口被纳入国家重点生态功能区转移支付范围。

（六）坚持以人民为中心，持续保障和改善民生

全年民生支出152.8亿元，同比增长18.9%，占地方一般公共预算支出的74.1%，省市民生实事全面完成，荣获中国全面小康特别贡献奖。精准脱贫深入推进。建立市区镇村四级脱贫攻坚责任制，投入财政扶贫资金1.03亿元，扶持建档立卡贫困户改造危房283户、转移就业817人、低保兜底保障619人、教育资助1967人、医疗救助386人，实现6个贫困村整村脱贫、8453人精准脱贫，超额完成年度脱贫攻坚任务。教育、医疗、文化、公共交通等社会事业加快发展。五源河学校等5所学校开学招生，新增中小学位6262个。扩大优质教育资源，与北师大附中、北大附中附小、人大附中、广东外语外贸大学附属学校等合作办学签约落地。进一步改善薄弱学校办学条件，四个区全部通过义务教育发展基本均衡县（市、区）国家验收。市属公立医疗机构全部实施“先看病、后付费”诊疗服务模式，累计受益人群5.4万人，垫付住院费用2.9亿元。广泛开展优生优育服务，积极推进两孩政策稳妥落地。新建村级文化室100个，歌舞诗《黎族家园》荣获第五届全国少数民族文艺汇演“剧目金奖”和“最佳舞美设计奖”。完成6家公交运营企业和1339辆公交车的整合；优化公交线97条。社会保障能力不断强化。实施积极的就业政策，城镇新增就业4.7万人，农村富余劳动力转移就业8386人。城乡居民基本医疗保险财政补助提高到420元，大病医疗保险实现全覆盖；低保救助37.7万人次，发放低保金1.1亿元。完成棚户区改造2.8万户、惠及8.5万人，保障性住房配租配售2922套。完成4万吨市级储备粮增储；落实省“菜十条”，出台“菜十六条”，“菜篮子”集团新建蔬菜保供种植基地6000亩，与广东湛江、山东寿光建立蔬菜应急调运机制，全省规模最大的公益性农副产品综合批发市场试营业，政府“平价菜”末端销售网点达265家，基本实现主城区大型超市、农贸市场、300户以上小区“三个全覆盖”，总体实现保供稳价，好于上年。社会治理不断加强。严厉打击违法犯罪，全面打响禁毒三年大会战，接报治安警情同比下降5%，去年首现下降拐点；刑事案件立案同比下降2.8%，破案率同比上升10%。获评第二届“全国创新社会治理优秀城市”。全面启动“七五”普法，建成公共法律服务中心，办理法律援助案件8552件。阳光信访、法治信访和责任信访有序推进。狠抓食药品、交通、电梯、旅游等重点领域的安全专项整治，获评全国安全生产监管工作先进单位，社会整体和谐稳定。

此外，统计、气象、地震、保密、档案、史志、科协、民族宗教、外事侨务、对台事务等工作取得新进展，工会、青少年、妇女儿童、老龄、残疾人、慈善等事业取得新进步。

（七）落实全面从严治党要求，强化政府自身建设

深入学习贯彻《关于新形势下党内政治生活的若干准则》和《中国共产党党内监督条例》，严格执行中央八项规定、省委省政府二十条规定和市委二十一条规定，驰而不息改进作风，政府自身建设明显加强。自觉接受人大法律监督、工作监督和政协民主监督，认真办理人大代表建议244件、政协委员提案307件，办结率均为100%。深入推进依法行政，全市38家单位权力清单和责任清单编制颁布；加强政府立法工作，提请市人大常委会审议法规议案2件，制定政府规章5件，依法行政工作连续八年被评为全省第一。强化审计监督，建立健全经济责任审计工作联席会议制度，集体约谈16家审计发现问题单位，全面抓好涉农资金问题整改。认真落实党风廉政建设主体责任，以《亮见》电视问政栏目为抓手，严厉查处庸政、懒政、怠政行为，问责干部111人；支持纪检监察和司法机关依纪依法查处腐败案件，全年共立案407件，处分449人。

各位代表！2016年是极不平凡的一年，面对经济下行的压力和“双创”的繁重任务，我们在全市经济结构调整、发展方式转变、生态环境只能更好的前提条件下，通过全市人民的共同努力，实现了经济社会的平稳较快增长，成绩来之不易，这得益于以习近平同志为核心的党中央的英明领导，得益于省委省政府和市委的坚强领导，得益于市人大、市政协及各位代表、各位委员的监督支持，得益于历届市四套班子打下的坚实基础，这是223万海口人民经过“双创”洗礼，团结一心、拼搏实干的结果！在此，我谨代表市人民政府，向全市人民，向人大代表、政协委员、各民主党派、工商联和社会各界人士，向驻市部队、武警官兵和公安司法干警，向关心支持海口的广大海外侨胞、港澳台同胞，表示崇高的敬意和衷心的感谢！

在肯定成绩的同时，我们也要清醒地认识到，当前还面临不少困难和问题：支柱性产业培育不够，核心竞争力不强；城乡发展不平衡，镇域经济发展滞后；城市规划设计水平有待提升，“双创”长效机制还需进一步完善；人才资源紧缺，交通、教育、医疗等基础设施和公共服务水平与人民群众期待还有一定差距；部分政府公务人员宗旨意识、纪律意识、担当意识仍需进一步加强，等等。对这些问题，包括各位代表、委员提出的其他问题，我们将高度重视，认真加以解决。

二、2017年工作安排

各位代表，今年我们将喜迎党的十九大和省第七次党代会的胜利召开，是贯彻落实市第十三次党代会的第一年，是我市抓规划、打基础的关键年，也是“双创”的决胜年，做好今年的政府工作意义重大。总体要求是：全面贯彻党的十八大和十八届历次全会精神，深入贯彻习近平总书记系列重要讲话精神，统筹推进“五位一体”总体布局和协调推进“四个全面”战略布局，牢固树立和贯彻落实新发展理念，坚持以提高发展质量和效益为中心，以推进供给侧结构性改革为主线，以“双创”为模式和动力，实施产业规划和城市规划双轮驱动，全力推进经济平稳健康发展，全力深化改革开放，全力加快城市更新，全力保障和改善民生，全面建设国际化滨江滨海花园城市，奋力谱写海南国际旅游岛建设主引擎新篇章。

主要预期目标是：全市生产总值增长7.5%左右，地方一般公共预算收入增长8%左右，固定资产投资增长16%左右，社会消费品零售总额增长8%左右，城乡常住居民人均可支配收入增长7.5%左右，居民消费价格涨幅控制在4%以内，城镇登记失业率控制在3%以内；全面完成省下达的节能减排目标任务。

为实现上述目标，我们将着力抓好以下八个方面的工作：

（一）积极扩大有效需求，确保经济平稳较快增长

加大投资力度。全年力争完成固定资产投资1475亿元，其中安排省重点项目98个、年度计划投资630亿元。推动万达文化旅游城、五源河文体中心、新海生态临港新城、疏港货运快速干道（G15海口段)等项目开工；加快美兰机场临空产业园、美兰国际机场二期扩建、马村港三四期、南渡江引水工程、铺前大桥等项目建设；确保江东大道二期、新海港二期等基础设施项目竣工，康迪电动汽车、威特电气、先声药业等一批生产性项目投产。加大引入社会资本力度，在棚改、生态治理、交通基础设施等领域谋划一批PPP项目，进一步强化规范运作、精准监管，全面提升PPP项目管理水平。坚持开展点对点敲门精准招商，争取引进一批支柱性产业项目、高新技术企业和龙头企业，特别是世界500强企业和“中字头”“国字头”等大企业、大集团，力争全年招商引资签约额度不低于1000亿元，为海口发展储备一批优质项目。

扩大消费需求。结合“互联网+”消费活动，大力推动和培育休闲旅游、健康养生、文化体育、信息、家政等新型热点消费，增加多层次、多样化消费产品供给。推动房地产业健康发展，推进商品房去库存，大力培育发展住房租赁市场，实施商品房全装修，引导房地产业向高端、特色、经营性转型。优化城市商业综合体布局，提升商贸物流中心建设水平，持续抓好城市便民生活圈建设，改造农村电子商务配送及综合服务网络，撬动大众消费的内生动力。

创新外贸服务。推进服务贸易创新发展试点，尽快在旅游、教育、医疗等9个领域引进一批服务项目和企业，力争实现服务贸易总额占全省一半以上。支持展示交易、融资租赁、保税仓储等外贸新业态发展，设立跨境电子商务综合示范区，重点建设跨境电商保税物流中心和进口商品展示直营中心，鼓励本地企业利用跨境电商开展出口业务。支持新能源汽车、医药等优势产品出口。积极引进跨国公司在海口设立区域总部。

（二）深化供给侧结构性改革，促进绿色特色产业成形成势

按照“一年打基础、三年成形、五年成势”要求，以“十二大产业”为主攻方向，实施更加精准的产业政策，培育壮大支柱性产业，着力构建绿色经济产业体系。

推动旅游业提档升级。“点线面”推动全域旅游。注重景区景点的规划设计，加快长影环球100等旅游吸引物建设，提升观澜湖度假区、雷琼世界地质公园、东寨港红树林生态旅游区等品牌影响力，力争5A级景区零突破；结合“美丽海南百千工程” 完成18个全域旅游镇墟改造，推动道路交通旅游化建设，构建便捷的旅游交通网络；大力实施“旅游+”战略，筹备召开旅游大会，推进旅游与文化体育、健康养生、农耕体验等产业融合发展，形成城市休闲、主题民宿、时尚运动等多元化旅游新业态，打造邮轮游艇、帆船帆板、休闲渔业等水上旅游新亮点，扩大高端旅游产品供给。构建安全有序的旅游服务体系。发展“智慧旅游”，启动旅游大数据平台建设，继续推进全域旅游综合执法体制改革，形成网状、立体式旅游治理机制。加大旅游营销投入和推广力度。细分目标市场，完善旅游市场开拓激励机制，增强外语、外币兑换等旅游公共服务能力，推出一批旅游景区套餐套票，运用“互联网+”等方式开展旅游精准营销，提升旅游产品“掌上能见度”和“世界能见度”。

加快新兴产业成长壮大。促进健康产业发展。筹建医疗健康大数据产业园、健康养生示范园和中医健康疗养基地，培育壮大一批健康养生企业。继续引进国内外优质医疗资源，推进与澳大利亚圆美集团合作框架协议落地，确保上海第六人民医院海口骨科与糖尿病医院如期建成营业。积极实施“互联网+”行动计划。建设“智慧海口”，加快数据中心、云计算、物联网和支撑平台建设，培育壮大软件业、游戏业、电商业，建设好复兴城创新创业园和海南数据谷。加快金融业发展。持续优化金融生态环境，支持金融机构做大做强，规划建设金融总部集聚区，重点发展以资本运作、财富管理和企业服务为主的金融总部经济，力争引进1-2家国内外金融机构。做大做优会展业，完善会展服务体系，提高会展专业化、国际化服务水平，引进一批国际会议和大公司年会，力争举办规模以上会展260场，综合收入突破百亿元。大力发展现代物流业，推动国家级物流枢纽中心城市建设，加快物流园区、保税物流、跨境电商物流建设，推动海陆空无缝衔接、高效联运，降低物流成本，争取林安物流区域总部落户。推动文体产业发展，培育发展旅游演艺、影视制作、动漫游戏等产业，放大冯小刚电影公社带动效应，力争中国（南方）足球训练基地项目挂牌，引进一批大型文体活动品牌，办好自行车竞技赛、“海丝杯”足球邀请赛等赛事。

强化创新驱动。大力推进“大众创业、万众创新”，建设一批“互联网+”创新创业示范基地、孵化（实训）基地。加大财政科技投入，支持企业、高校、科研院所开展技术攻关和核心产品研发，着力引进国家级研究机构、重点实验室落户。强化园区平台支撑作用，研究推出“飞地政策”，引导低碳制造企业、龙头企业和高新技术企业向园区集聚集约发展。力争新培育和引进高新技术企业30家以上，孵化、认定科技创新型企业5家以上。研究制定一致性评价支持政策，帮助医药企业转型发展，高水平建设“新药谷”。大力发展海洋经济，在海洋生物、海洋装备制造、深海资源等方面谋划一批重大项目，探索建设1-2个海洋牧场示范区，积极申报国家级海洋经济创新发展示范市。

（三）实施城市更新计划与行动，打造海口“双创”升级版

今年是“双创”决胜之年，在坚持工作标准不变、力度不减、确保“双创”夺牌的基础上，加快城市更新，全面建设国际化滨江滨海花园城市。

强化城市规划设计。按照“一江两‘岸’、东西双港驱动、南北协调发展”的总体思路，坚持“产城融合、港城融合、城乡融合”理念，围绕海绵城市、花园城市建设，认真编制《海口城市更新计划与行动》，系统开展生态修复、城市功能、资源统筹、公共设施、城市品位、道路交通、历史文化等专项规划。完成海甸溪两岸和新海港区、秀英港区的规划设计，全面启动《湿地保护与修复工作实施方案》三年计划，做好红树林湿地、羊山湿地、火山风貌文章，使城市整体风格更加和谐、协调。

加快城市更新改造。优化棚改政策，推进棚改与加快城市更新、调整产业结构、改善群众生活、消化房产存量相结合，提升棚改综合效益和城市价值。继续推进城市“五网”基础设施建设，加大主城区主次干道改造，优化交通道路网络和公交体系，开展轨道交通前期工作。加快地下综合管廊建设。新建江东水厂，扩建永庄水厂，完善供水管网配套，提升城市供水保障能力。持续加大城市绿化彩化亮化美化力度，全面完成国兴大道、滨海大道等主干道和美兰机场、火车站等重点区域的景观改造工程。健全城市公共服务设施，开工建设“市民中心”、市图书馆、博物馆，加快国兴“双子塔”、海口湾等城市“新地标”“新客厅”建设，争取启动三江湿地公园、新海生态临港新城、南渡江两岸景观提升等首批15个城市更新工程。

强化城市治理。进一步拓展“12345”热线服务内容，建立健全“集中受理、分类处置、各方联动、限时办结、回访答复、监督考评”的工作机制，把市民游客诉求有效解决在基层。深化“公安+城管”联合执法，探索建立旅游、国土、环保、工商、食药监等多部门综合执法机制，持续开展车辆超载、占道经营、交通秩序、环境卫生等专项整治，加强巡查督查、实施严管重罚；支持社团组织参与城市治理，实现“双创”管理长效化、常态化。

提升城市文化内涵。夯实国家历史文化名城底蕴，扎实推进骑楼老街保护和改造，丰富经营业态。全面实施“琼台复兴计划”，保护修缮琼台福地、琼台书院、鼓楼等古迹，弘扬冼夫人文化，支持“府城元宵换花节”申报国家级非物质文化遗产。加大龙华东谭、琼山包道等13个“中国传统村落”和火山民居古村落保护。弘扬社会主义核心价值观，加强青少年思想道德教育，创新发展乡贤文化，完善“诚信海口”“公益海口”建设，建立学雷锋志愿服务长效机制，持续开展精神文明创建活动。

做好城市经营文章。强化政府主导土地一级开发，结合“多规合一”的实施、城市规划的调整，完善土地征收和土地储备机制，重点做好重大产业项目、重大基础设施、重大生态修复工程和重大城市开发片区等周边土地的收储。开展国有建设用地清理，加大闲置土地清理盘活力度，建立项目综合评估和退出机制，更加注重引进高附加值、高利税、高成长性、高科技含量项目，提升土地综合效益。全面清理城市资源、资产，针对不同类型的资产实施相应的资产证券化，切实提高城市经营效益。

（四）深入开展脱贫攻坚，全力推进“三农”工作

坚决完成脱贫任务。把脱贫攻坚作为头等大事和第一民生工程，按照“两年脱贫攻坚，三年巩固提升”的总体目标，突出问题导向，强化动态管理，瞄准建档立卡贫困人口，加大项目、资金、政策、培训夜校等投入力度，全面实施交通基础设施扶贫六大工程建设，确保5个贫困村整村脱贫、2850人精准脱贫，全面完成脱贫攻坚任务。

大力发展现代农业。围绕“一镇一业、一村一品”，推进农业品牌化建设，抓好农产品“三品一标”认证，扩大十大农业品牌种植规模。加大对龙头企业、农民专业合作社和特色农业的扶持力度，重点抓好蔬菜、兰花、石斛等产业基地建设。大力发展生态循环农业，新建3-4个生态循环农业示范基地。深化“无疫区”建设，加强农业科技和农产品质量安全体系建设。推动农业产业链延伸升级，鼓励农产品加工，大力发展农村民宿经济。

促进农民增收。实施农民增收计划，争取今年农民收入增长8%以上。加大农民培训力度，提升农业产业化经营水平，重点抓好10个合作示范社建设，培养和推广一批电商示范户，增加农民经营性收入。做好劳动力与企业用工的有效对接，农村富余劳动力转移就业6000人以上，增加农民工资性收入。推进农民土地经营权入股，发展多种形式股份合作，增加农民资产性收入。

夯实农村基础工作。稳步推进农村集体产权制度改革，加快农村土地确权颁证，实施农村土地集体所有权、承包权、经营权“三权分置”，下力气解决农村土地抛荒问题。深化村务公开，强化农村集体资金、资产、资源管理。加强农村“五网”、垃圾转运、污水处理等基础设施建设，加快江东等10个输变电工程建设，全面完成19个中心村农网升级改造，完善农村小型水利工程管理体制，抓好农田水利整治修复。提高农村教育、医疗等公共服务水平，改善农村生产生活条件。

（五）加大改革开放，进一步释放发展活力

继续深化重点领域改革。扎实推进“多规合一”落地实施。组建市规划委员会，建立“编、审、管、监”一体化机制体制，形成与“多规合一”相配套的政策法规体系，搭建以“多规合一”为纲的综合信息平台，确保“一张蓝图干到底”。深化“放管服”改革。探索设立市行政审批局，积极推进审批提速，压缩审批时间25%以上，削减审批环节15%以上。总结提升和推广“极简审批”，大力推行工程建设项目多评合一、多审合一、多图联审、联合验收等模式。加强事中事后监管，加快建设企业信用信息共享平台，构建跨部门执法联动响应及失信约束机制，创建社会信用体系建设示范城市。加快国企国资改革步伐。做大做强国有企业，加快市属国有企业重组整合，选择1-2家企业进行混合所有制改革试点，探索建立以管资本为主的监管体制机制。深化公立医院综合改革。探索建立医疗管理集团，推进现代医院管理制度建设。此外，稳步推进国有林场、不动产登记和农场属地化管理等改革工作。

扩大开放合作。加快“一带一路”战略支点城市建设，深化与泛珠、北部湾区域合作，完善与湛江的协作协调机制，加快琼州海峡港航资源整合，推进琼州海峡经济带发展。加快“海澄文”综合经济圈建设，推进规划、基础设施等“六个一体化”，启动琼北滨海旅游公路等交通工程建设，争取海口新区年内获得国家批复。制定出台吸引外资的优惠政策，保护外商投资企业及其投资者权益，主动融入经济全球化。扩大国际交流与合作，探索与“一带一路”沿线国家互办文化年、旅游年、艺术节。

（六）推进生态文明建设，持续巩固提升生态优势

牢固树立“绿水青山就是金山银山”的强烈意识，坚持生态立市不动摇，让生态环境越来越好。

深化生态环境专项整治。强化“山水林田湖生命共同体”生态修复和湿地保护。加大对东寨港红树林、羊山湿地等生态敏感区保护，继续实施“绿化宝岛大行动”，完成植树造林1万亩，森林覆盖率保持在38%以上。推进水体整治。建立健全市区镇（街）三级河长制，开展海绵城市建设试点，探索实施生态化水体治理方式，完善城市污水处理设施和配套管网，加强集中式饮用水水源土地的收储和保护，注重水体自然景观提升，打造人水相亲的舒适环境。持续强化海岸带保护与开发。启动海洋生态系统修复工程，开展蓝色海湾整治行动，重点加强海口湾、秀英港等海湾港口的生态修复，确保90%近岸海域海水水质达到二类以上标准。深入开展土壤环境质量调查。加大南渡江非法采沙整治，加强矿坑修复与治理，保持土壤环境质量总体稳定。继续开展整治违法建筑专项行动。年内拆违200万平方米，统筹做好拆后垃圾清运、土地利用、环境修复，加强乡镇和村庄规划建设管理，建立健全打违控违长效机制，扎实推进“无违建”创建工作。开展大气污染防治专项行动。加强工地扬尘、汽车尾气等治理，确保空气质量在全国74个重点城市中继续保持第一。

健全生态文明建设制度体系。实行最严格的环境保护制度，实行领导干部自然资源资产离任审计和生态环境损害责任终身追究制。落实环保机构监测执法垂直管理改革。积极推行环境污染和治理第三方评估，建立多元化的资金投入机制。完善环境标准体系，加强生态环境监测网络建设，建立健全环保数据共享和信息公开披露制度。开展绿色园区、绿色工厂等创建活动，加大新能源汽车应用推广力度，鼓励市民绿色出行，积极倡导和推行绿色生产和绿色生活方式。

（七）积极回应百姓诉求，持续保障和改善民生

始终以人民为中心，以更大的决心和力度改善民生，办好省市为民办实事事项，让改革发展成果惠及更多群众，人民生活更加幸福美满。

协调社会事业发展。优先发展教育，强化师资力量，实施特岗教师和乡村教师定向培养计划，加快“全面改薄”和“10+2” 项目建设，力争全年新增中小学学位6000个以上，继续大力引进国内外优质教育资源，推进人大附中、北师大附中、北大附中附小等合作办学项目，办好中职教育和特殊教育。建设“健康海口”，不断提高医疗服务质量，完善社区医疗卫生服务体系，推进乡村卫生院（室）标准化建设，落实医师多点执业政策，实施120院前急救服务和区域二级医疗机构能力提升工程；加强生育全程基本医疗优质服务，促进人口均衡发展。加快推进公共文化服务体系建设，推进区级文化馆、村级文化室建设，完善一批居民小区文体设施，加快推进城区电子阅报屏和乡村数字农家书屋建设，深入开展全民健身运动，丰富群众文娱体育生活。

提高社会保障水平。实施更加积极的就业政策，做好高校毕业生、退役军人就业工作，建立低保与就业联动机制，城镇新增就业3万人。提高城乡居民社会养老保险参保率，扩大基本医疗保险保障范围，全面开展重特大疾病医疗救助，继续深化机关事业单位养老保险制度改革。严格落实“米袋子”“菜篮子”责任制，推进省市共建粮食物流园区，提升市菜篮子集团在生产、流通、销售各环节的运营和调节能力，实现保供稳价。

强化社会综合治理。深化“平安海口”建设，完善立体化社会治安防控体系，实施“八严”工程，坚决打赢禁毒三年大会战。加强产品质量、食品药品安全监管，积极创建国家食品安全城市。全面落实安全生产责任制，坚决预防和遏制重特大安全事故。深入开展“七五”普法，做好信访积案化解，强化网络舆情监测和应急处突。

全力支持国防建设，推动军地融合发展。充分发挥工会、共青团、妇联、残联等人民团体桥梁纽带作用。继续做好统计、人防、地震、气象、档案、慈善、史志、保密、外事侨务、对台、民族宗教等各项工作。

(八)加强政府自身建设，优化海口发展环境

打造廉洁高效的政务环境。坚决维护以习近平同志为核心的党中央权威和集中统一领导，坚决落实全面从严治党要求，认真贯彻《关于新形势下党内政治生活的若干准则》和《中国共产党党内监督条例》，切实强化“四种意识”，推动党中央国务院、省委省政府和市委各项决策部署得到不折不扣贯彻落实。严格执行市人大及其常委会的决议、决定，强化政协民主协商，自觉接受人大监督、政协监督、社会监督和舆论监督。坚持依法行政，落实政府法律顾问制度，完善政府立法工作机制，提升政府决策法制化水平。实施“互联网+政务服务”，最大限度推进政府服务信息共享，提升“大数据”综合服务能力和精细化管理水平，不断提高行政效能。完善市直机关绩效管理办法，建立正向激励和容错纠错机制。落实党风廉政“一岗双责”，坚持把纪律和规矩挺在前面，不断扎紧制度的笼子，加强监察审计和督査考核，加大行政问责力度，严惩懒政怠政。

优化投资发展的营商环境。进一步强化投资服务保障，固化“百日大行动”长效机制，做好土地、林地、用海、融资、供电、供水、供气等要素保障服务，为投资项目顺利推进保驾护航。在推进棚改项目、重大基础设施建设的同时，注重完善重大项目、重点园区的基础设施配套，合理布局教育、医疗等公共服务资源。完善政银企合作对接机制，落实各项扶持中小微企业发展的政策措施，解决好融资难、融资贵的问题，降低企业运营成本，全力打造“全国一流、海南领先”营商环境。加强对重点工程、重大项目和涉农资金等重点领域的廉政风险防控，努力构建“亲”“清”新型政商关系。

营造吸引人才的社会环境。推进“人才强市”战略，出台鼓励人才引进、培养、使用的政策措施，积极引进创新创业领军人才、产业发展紧缺人才、基层急需技能人才和党政资政人才。扶持建立各类“候鸟”人才社会组织，争取引进一批“名师名医名家名匠”专业人才。开展“一站式”人才服务，着力解决人才后顾之忧，在住房保障、户籍迁移、家属就业、子女入学等方面提供全方位服务，努力营造尊重劳动、尊重知识、尊重人才、尊重创造的社会氛围。

各位代表，建设海口幸福家园是全市人民的期待和重托。让我们更加紧密地团结在以习近平同志为核心的党中央周围，在省委省政府和市委的坚强领导下，不忘初心，继续前进，撸起袖子加油干，以优异成绩向党的十九大和省第七次党代会献礼！
